# Role of bacteriophages in STEC infections: new implications for the design of prophylactic and treatment approaches

**DOI:** 10.12688/f1000research.3718.2

**Published:** 2014-08-26

**Authors:** Jaime H. Amorim, Manuel E. Del Cogliano, Romina J. Fernandez-Brando, Marcos F. Bilen, Monica R. Jesus, Wilson B. Luiz, Marina S. Palermo, Rita C.C. Ferreira, Esteban G. Servat, Pablo D. Ghiringhelli, Luis C.S Ferreira, Leticia V. Bentancor

**Affiliations:** 1 Department of Microbiology, Institute of Biomedical Sciences, University of São Paulo, São Paulo, 05508-060, Brazil; 2 Laboratorio de Ingeniería Genética y Biología Celular y Molecular, Universidad Nacional de Quilmes, Buenos Aires, 1876, Argentina; 3 BioSur, Ciudad Autónoma de Buenos Aires, Buenos Aires, 1406, Argentina; 4 Laboratorio de Patogénesis e Inmunología de Procesos Infecciosos, Instituto de Medicina Experimental (IMEX) (CONICET), Academia Nacional de Medicina, Buenos Aires, 1425, Argentina

## Abstract

Shiga toxin (Stx) is considered the main virulence factor in Shiga toxin-producing *Escherichia coli* (STEC) infections. Previously we reported the expression of biologically active Stx by eukaryotic cells *in vitro *and *in vivo* following transfection with plasmids encoding Stx under control of the native bacterial promoter ^1,2^. Since *stx* genes are present in the genome of lysogenic bacteriophages, here we evaluated the relevance of bacteriophages during STEC infection. We used the non-pathogenic *E. coli* C600 strain carrying a lysogenic 933W mutant bacteriophage in which the *stx* operon was replaced by a gene encoding the green fluorescent protein (GFP). Tracking GFP expression using an *In Vivo* Imaging System (IVIS), we detected fluorescence in liver, kidney, and intestine of mice infected with the recombinant *E. coli* strain after treatment with ciprofloxacin, which induces the lytic replication and release of bacteriophages. In addition, we showed that chitosan, a linear polysaccharide composed of d-glucosamine residues and with a number of commercial and biomedical uses, had strong anti-bacteriophage effects, as demonstrated at *in vitro* and *in vivo *conditions. These findings bring promising perspectives for the prevention and treatment of haemolytic uremic syndrome (HUS) cases.

## Introduction

Infections by Shiga toxin-producing *Escherichia coli* (STEC) strains are a serious public health concern, resulting in diarrhea, hemorrhagic colitis, and haemolytic uremic syndrome (HUS).

Stx is the main virulence factor in STEC strains. The *stx* gene is present in the genome of prophages, which are similar to the bacteriophage lambda found in the lysogenic form of various *E. coli* strains. Previously we reported that the native promoter of the Stx-encoding gene can drive expression of the toxin in eukaryotic cells in both *in vivo* and *in vitro* conditions ^1, 2^.

Many questions remain unanswered with regard to the mechanism by which STEC infection causes HUS. In particular, we are interested in understanding how Stx enters the systemic circulation and why only very small numbers of bacteria are sufficient to induce HUS in humans ^3^.

Based on our previous observations that the native *stx* gene promoter is active in host cells, we seek to understand the role bacteriophages play in the pathogenesis of STEC strains. Recently, it was reported that bacteriophages carrying the *stx* gene are required for the development of HUS in the murine model ^4^. Our hypothesis is that eukaryotic host cells are transduced with and/or infected by Stx-encoding bacteriophages, leading to *in vivo* dissemination after entry in. This would also explain why very small numbers of bacteria are sufficient to cause HUS.

In order to test whether bacteriophages are responsible for the induction of HUS, we used an anti-bacteriophage agent to inactivate them. Chitosan, a linear polysaccharide polymer obtained after the deacetylation of chitin, the structural element in the exoskeleton of crustaceans, possesses strong antimicrobial activity against several pathogenic microorganisms ^5^. Its antiviral activity was reported on the bacteriophage c2, which infects *Lactococcus* strains, and on bacteriophage MS2, which infects *E. coli* ^6^, without significantly affecting the growth of the bacterial strains ^7^. In order to test our hypothesis, which would make Stx-encoding bacteriophages a new target for prevention and treatment of STEC infections; we used chitosan as an anti-bacteriophage agent both *in vitro* and *in vivo*.

For that purpose we employed recombinant phages in which the Stx-encoding genes were replaced by the gene encoding the green fluorescent protein (GFP). The results demonstrated that STEC phages can systemically disseminate in different mouse tissues and organs after delivery directly into the stomach of mice. In addition, with the present results we demonstrated that chitosan has strong inhibitory effects on STEC bacteriophage as demonstrated under *in vitro* and *in vivo* conditions.

## Materials and methods

### Strains

The *E. coli* C600ΔTOX:GFP strain is a lysogenized C600 strain carrying the 933W bacteriophage in which the *stx* gene was replaced by the *gfp* sequence (ϕΔTOX:GFP) ^8^. The bacterial strain was generously provided by Dr. Alison Weiss. The enterohemorrhagic *E. coli* (EHEC) EDL933W strain (ATCC 43895) is lysogenic for the wild-type bacteriophage from which ϕΔTOX:GFP was obtained. *E. coli* Y 1090 strain was used in the bacteriophage titration assay (ATCC 37197).

### Transduction of eukaryotic cells

BHK-21 cells (Syrian hamster kidney fibroblasts from the American Type Culture Collection) cells were grown on 12-well plates (Nunc) in complete medium (10% fetal bovine serum in DMEM medium, Gibco, USA) for use in the transduction assay. Phages (ϕΔTOX:GFP), at a multiplicity of infection (M.O.I) equal to 1, were added to BHK-21 cells spread the day before on 12 wells plate (Nunc). BHK-21 cells were counted with a Neubauer camera, and the bacteriophage titers were measured as described below. Transduction of BHK-21 cells was enhanced by centrifugation at 1,000 × g for 10 min at room temperature as previously reported ^1^. After incubation at 37°C for 3 hours, the phage-containing medium was removed. Cells were washed twice with phosphate buffered saline (PBS) and then incubated in complete medium (DMEM, Gibco, USA). Twenty four hours post-transduction, cells were washed with PBS, harvested by Trypsin-EDTA incubation and centrifuged at 2,655 × g for 15 minutes. DNA was harvested from pellets after incubation for 5 minutes at 98°C in lysis solution (Tris pH8 50 mM, SDS 2%, Triton-X100 5%) and the harvested DNA was used for PCR. Primers: Up-R 5´CCGCTCGAGACTAGTGCAAAAGCGAGCCTGGTAAATAAATATG3´; Up-D 5´GGAATTCCATATGCTCGTTGAGGCATATGAAAATCAGAC3´. The reaction was run in a Eppendorf Termocycler at an initial 92°C for 120 seconds and then at 92°C for 20 seconds and 60°C for 20 seconds and 72°C for 120 seconds for 35 cycles using primers giving a fragment of 1310 bp on the upstream region of *gfp* gene into the bacteriophage genome.

### Bacteriophage induction

The *E. coli* C600ΔTOX:GFP strain was grown in Luria Broth (LB) plus 10 mM CaCl _2_ and chloroamphenicol (Sigma) (15 μg/ml final concentration) overnight (ON) at 37°C under agitation. The ON culture was diluted to OD _600nm_ = 0.1 in LB plus 10 mM CaCl _2_ and chloramphenicol (Sigma) (15 μg/ml final concentration). Induction was carried out by adding ciprofloxacin to a final concentration of 40 ng/ml ^9^. Bacteria were incubated for 6 hours at 37°C under agitation. Cultures were then centrifuged at 5000 rpm for 15 minutes. The bacteriophage-containing supernatant was filtered with 0.2 μm filters and kept at 4°C until the titration assay was performed.

### Titration assay

*E. coli* strain (ATCC 37197) was grown in LB plus ampicillin overnight at 37°C under agitation. The culture was diluted 1:100 in LB plus ampicillin and incubated for 2 additional hours at 37°C under agitation. At the end of the incubation, 500 μl samples of the *E. coli* strain were incubated with 5, 50 and 100 μl of a suspension containing bacteriophages for 30 minutes at room temperature. At the end of this incubation, 3 ml of Top Agar (Tryptone 1%; NaCl 0.5%; Agar 0.7%) plus CaCl _2_ (10 mM final concentration) was added, and plated on LB-Amp agar plates. Plates were incubated at 37°C and lysis plaques were visually counted.

### Bacteriophage inactivation assay

The ϕΔTOX:GFP phage was incubated with chitosan (Sigma 448877) at a final concentration of 5 mg/ml in phosphate buffer 10 mM, at Ph = 7 for 10 minutes at room temperature, and the bacteriophage titers were measured as described in titration assay section. Chitosan was also used in the bacteriophage induction assay described above. Chitosan was added 2 and 4 hours post-induction and bacteriophage titers were analyzed at 6 hours post-induction.

### Mice

BALB/c and DBA-2 mice were bred in-house at the animal facility of the Microbiology Department of the Sao Paulo University, Brazil. The protocol was approved by the Ethics Committee on Animal Experiments of the Institute of Biomedical Sciences (Protocol number 106), University of São Paulo. Male mice aged 6 weeks (18 to 20 g) were used for the *In Vivo* Imaging System (IVIS). Immature male and female DBA-2 mice (17–21 days of age, approximately 8–11 g body weight) were used immediately after weaning for the infection assays with EDL933W strain (n = 4). Mice were maintained under a 12-hour light-dark cycle at 22 ± 2°C and fed a standard diet and water *ad libitum*.

### Ethics statement

The experimental protocol of this study followed the ethical principles for animal experimentation adopted by the Brazilian College of Animal Experimentation (COBEA) and was approved by the Ethics Committee on Animal Experiments of the Institute of Biomedical Sciences (Protocol number 106), University of São Paulo, in accordance with the principles set forth in the Guide for the Care and Use of Laboratory Animals (National Institutes of Health, 1985).

### EHEC infection

Immature male and female DBA-2 mice (17–21 days of age, approximately 8–11 g body weight) were used immediately after weaning for the infection assays (n = 4). *E. coli* EDL933W strain was used for the mouse infection experiments following the protocol previously reported by Brando and collaborators ^9^. Briefly, the *E. coli* EDL933W strain was grown in Tryptic Soy Broth (TSB, DIFCO, BD) overnight at 37°C. The culture was centrifuged at 14,000 rpm for 15 minutes and the bacterial pellet washed twice in phosphate buffered saline (PBS). Bacterial cells were suspended to a final concentration of 3 × 10 ^13^ CFU/ml. The bacterial suspension (100 μl) was delivered directly into the stomach of mice after 8 hours of food starvation, via a gavage needle. After 4 hours of ingesting the bacterial suspension, mice were given food and water. Control animals received 100 μl of sterile PBS. Survival was observed for one week. Both groups were composed by 4 animals.

### *In vivo* chitosan protective effects

Immature male and female DBA-2 were infected with the *E. coli* EDL933W strain, as described above, and treated with 100 μl of a chitosan solution at a concentration of 5 mg/ml (500 μg of chitosan per mouse) orally administered 2 hours after infection and survival was recorded. Chitosan effects were also measured. -month old BALB/c mice orally infected with the *E. coli* C600ϕΔTOX:GFP strain. *In vivo* bacteriophage induction was carried out with ciprofloxacin as previously described by Zhang and collaborators ^9^. Two 2 hours after induction with ciprofloxacin, 100 μl of the chitosan solution was administered orally to the mice and GFP dissemination by IVIS was analyzed.

### *In Vivo* Imaging System (IVIS)

Two-month old BALB/c mice were orally infected with the *E. coli* C600:ϕΔTOX-GFP strain. Briefly, bacterial cells cultivated overnight in LB medium were washed with phosphate buffered saline (PBS), centrifuged again and suspended in a 20% sucrose to have a concentration of 1 × 10 ^10^ CFU. Mice were inoculated orally with 10 ^9^ bacterial cells and *in vivo* bacteriophage excision was carried out as described by Zhang and collaborators ^9^. Mice were submitted to euthanasia with CO _2_ inhalation 24 hours later. Blood, spleens, kidneys, lungs, brains, intestines, hearts and livers were harvested by surgical removal and kept in PBS solution for evaluation of GFP expression. GFP was excited at 465nm and detected at 510nm. Mice were analysed in a living Imaging 4.3.1 Calipter model (Life Sciences).

### Statistical analysis

Statistical significance between treatments and controls was analyzed using the Prism 5.0 software (GraphPad Software), and the corresponding *P* values are indicated in the figures. Data correspond to means ± standard errors of the means (SEM) for individual mice. Statistical differences were determined using the one-way analysis of variance (ANOVA).

## Results

### Induction of ϕΔTOX:GFP by ciprofloxacin and chitosan anti-bacteriophage effects

**Figure 1.**
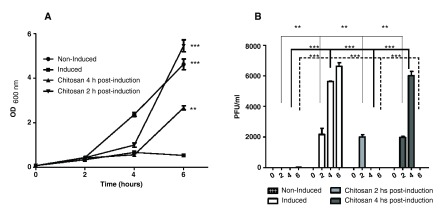
Induction of ϕΔTOX:GFP by ciprofloxacin and effect of chitosan *in vitro*. **A**. Growth curve: the *E. coli* C600ΔTOX:GFP strain was induced with ciprofloxacin and the optical density was measured at 600nm at 0, 2, 4 and 6 hours after induction. Non-induced culture of *E. coli* C600ΔTOX:GFP strain was used as control. Chitosan was added at 2 or 4 hours after induction. **B**. Bacteriophage ϕΔTOX:GFP titers: bacteriophage titers were determined at 0, 2, 4 and 6 hours post-induction. Chitosan was added at 2 or 4 h post-induction. * *p*<0.05.

Lytic induction was triggered in the *E. coli* C600ΔTOX:GFP strain using ciprofloxacin ^9^. We observed a significant decrease in the optical density of the bacterial culture after addition of the antibiotic and the release of phages into the culture supernatant ( Figure 1, panel A and B). The bacteriophage titers were determined at different time points after lytic induction and a significant increase in the number of viable bacteriophages was observed after induction ( Figure 1, panel B). The effect of chitosan as an anti-bacteriophage agent was also examined. To this aim, we added chitosan to the bacterial culture 2 or 4 hours post-induction and we observed the complete inactivation of the ϕΔTOX:GFP without measurable toxic effects to the bacterial strain ( Figure 1, panels A and B).

### Transduction of mammalian cells with ϕΔTOX:GFP

**Figure 2.**
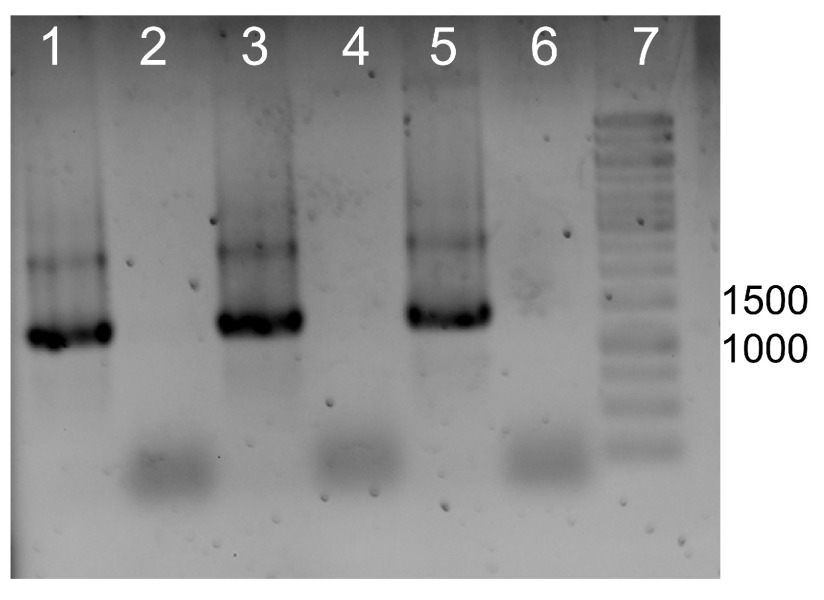
Detection of ϕΔTOX:GFP DNA in transfected mammalian cells. **A**. PCR on DNA extracted from BHK-21 cells: 24 hours after transduction, BHK-21 cells were washed and treated with Trypsin-EDTA solution. DNA was extracted and PCR was performed. Lane 1: Cells transfected with ϕΔTOX:GFP. Lane 2: Cells transfected with ϕΔTOX:GFP previously treated with chitosan. Lane 3: Cells transfected with ϕΔTOX:GFP previously treated with DNAse. Lane 4. Untreated cells. Lane 5. Positive PCR control (ϕΔTOX:GFP DNA). Lane 6. Negative PCR control. Lane 7. 1 kb ladder (Invitrogen).

We previously reported the capacity of ϕΔTOX:GFP to transduce macrophages *in vitro*. To further evaluate the ability of chitosan to inhibit bacteriophage transduction, BHK-21 cells were transduced for 3 hours with ϕΔTOX:GFP, ϕΔTOX:GFP plus chitosan or ϕΔTOX:GFP previously treated with DNAse. Addition of DNAse would eliminate any free bacteriophage DNA in the bacterial lysates. As shown in Figure 2, ϕΔTOX:GFP DNA was detected by PCR in exposed mammalian cells, confirming that the virus was proficient to transduce this cell line. Similar results were also obtained in cells exposed to bacteriophages treated with DNAse ( Figure 2). However, no phage DNA was detected when BHK-21 cells were infected with ϕΔTOX:GFP incubated with chitosan, confirming the inactivating action of chitosan on ϕΔTOX:GFP ( Figure 2).

### GFP detection in mice inoculated with the lysogenic *E. coli* C600Δ *TOX:GFP strain*

**Figure 3.**
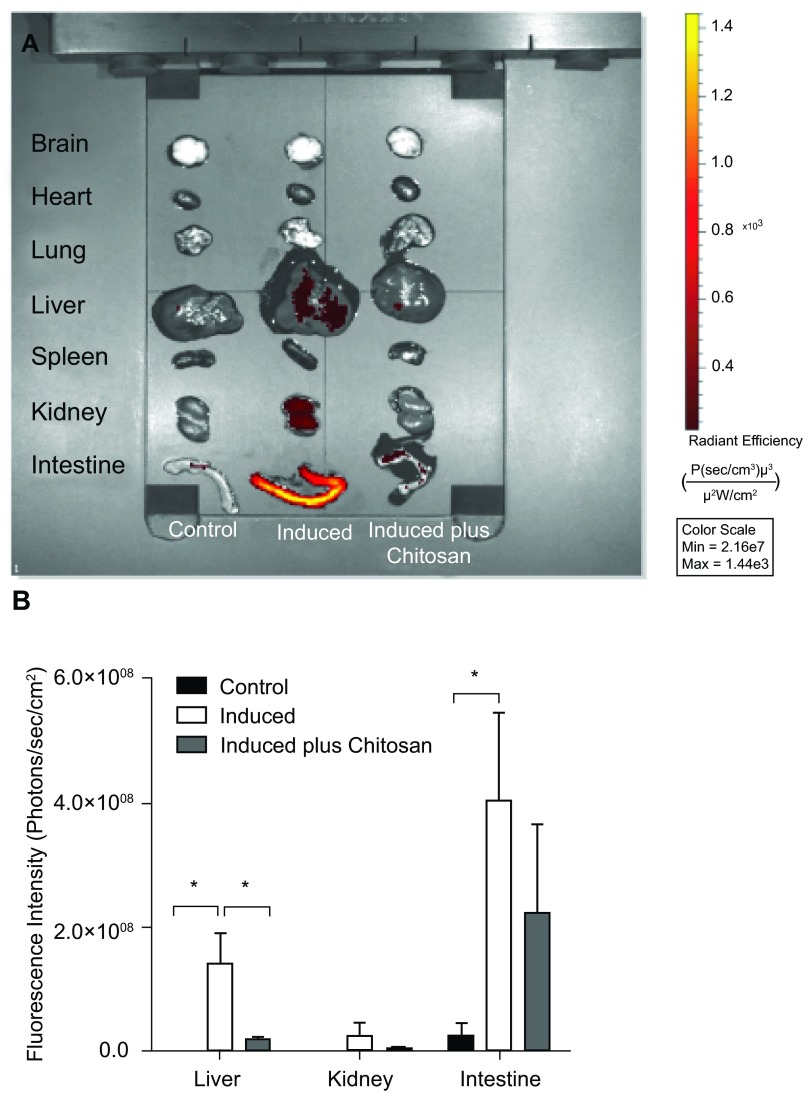
Detection of *in vivo* GFP expression in mice infected with the lysogenic *E. coli* C600ΔTOX:GFP strain using *In Vivo* Imaging System (IVIS). **A**. IVIS Representative image: ciprofloxacin was administered 2 hours post-infection to induce ϕΔTOX:GFP *in vivo*. Mice were treated with chitosan 2 hours after bacteriophage induction. All mice were sacrificed 24 hours post-infection and brains, hearts, lungs, livers, spleens, kidneys and intestines were harvested and analyzed by IVIS. Fluorescence intensity was recorded as photons/sec/cm ^2^, and the signal intensity represents the amount of GFP present. **B**. Graphic of fluorescence intensity on GFP-positive organs. Four animals per group were analyzed and the fluorescence intensity was quantified using Living Imaging 4.3.1 in Calipter Life Sciences.

To demonstrate the *in vivo* dissemination of ϕΔTOX:GFP, mice were infected with the lysogenic *E. coli* C600ΔTOX:GFP strain followed by gastrointestinal administration of ciprofloxacin. In order to evaluate the effect of chitosan *in vivo*, a group of mice was administered with chitosan 2 hours post-induction and a control group of uninfected mice was evaluated for auto-fluorescence background control in each organ. One day after infection, organs were harvested and examined for GFP expression. As shown in Figure 3, GFP was detected in the intestine, liver and, to a lesser extent, kidney of mice orally infected with the lysogenic *E. coli* C600ΔTOX:GFP strain and treated with ciprofloxacin. Remarkably, administration of chitosan 2 hours after infection caused a sharp decrease in GFP detection in organs of orally infected mice ( Figure 3, panels A and B). Moreover, positive detection of phages was observed in intestine homogenates and blood samples of infected mice after ciprofloxacin induction (data not shown). These results indirectly demonstrate that ϕΔTOX:GFP is released by the lysogenic bacterial *E. coli* strain and systemically spread and transduce cells in different mouse organs and tissues after oral infection and lytic induction. Another possibility is that, the bacteriophage could be taken by pinocytosis by eukaryotic cells, and, once inside the cell, GFP or Stx2 are expressed.

### Effect of chitosan on the mortality of mice orally inoculated with the EHEC EDL933W strain

**Figure 4.**
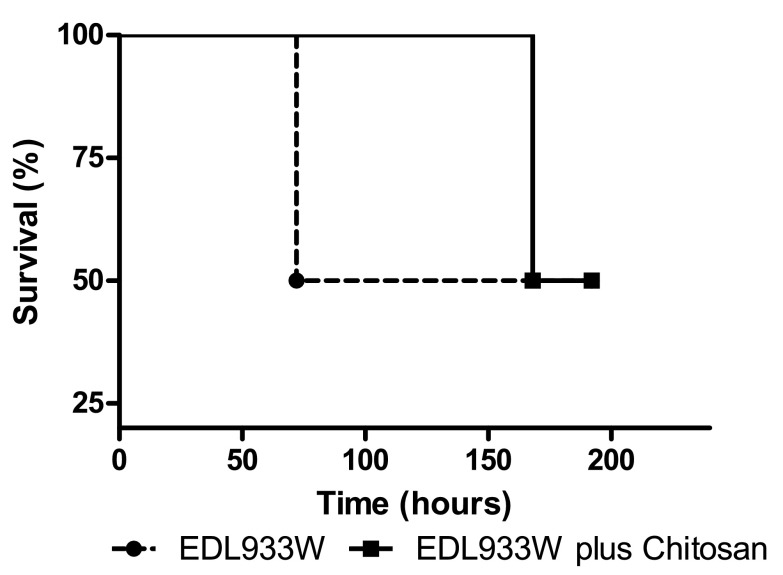
Treatment with chitosan delays death of mice infected with the EHEC EDL933W strain. Mice were infected orally with the EHEC EDL933W strain. Controls did not receive chitosan (dots and broken line) and the experimental group received chitosan 2 hours post-infection (square and fill line). Survival rates were followed for one week.

In order to evaluate the *in vivo* effect of chitosan during the infection process, mice were intragastrically infected with a wild-type EHEC EDL933W strain, based on the model described by Brando and collaborators ^10^. Another mouse group was also treated with chitosan, intragastrically administered 2 hours post-infection, and survival was followed for one week. Partial protection was observed in mice treated with chitosan as demonstrated by the delay in the death time ( Figure 4). Mice infected with the EHEC EDL933W strain died 72 hours post-infection while mice infected with the same strain and subsequently treated with chitosan died 168 hours after infection.

Induction of ϕΔTOX:GFP strain by ciprofloxacin and chitosan effect Induction of ϕΔTOX:GFP by ciprofloxacin and effect of chitosan *in vitro*. A. Growth curve: the *E. coli* C600DTOX:GFP strain was induced with ciprofloxacin and the optical density was measured at 600 nm at 0, 2, 4 and 6 hours after induction. Non-induced culture of *E. coli* C600DTOX:GFP strain was used as control. Chitosan was added at 2 or 4 hours after induction. B. Bacteriophage ϕΔTOX:GFP titers: bacteriophage titers were determined at 0, 2, 4 and 6 hours post-induction. Chitosan was added at 2 or 4 h post-induction. *p<0.05.

## Discussion

Lambda bacteriophages are used as carriers in gene transfer and vaccine delivery experiments based on the capacity to *in vivo* transduce mammalian cells ^11^. Tyler and collaborators recently showed that prophage induction is required for renal disease and lethality in the EHEC mouse model, suggesting that free bacteriophages encoding Stx may play a direct role in the disease ^4^. Our results give a further support to that hypothesis and help understand why only small numbers of bacteria are usually capable to induce HUS in humans ^3^. If bacteriophages are induced in the gastrointestinal tract, infect different host cells, and promote Stx expression, a reduced number of bacteria would suffice to cause significant damage.

In previous reports, we showed that the native phage promoter controlling Stx expression is active in eukaryotic cells both *in vitro* ^1^ and *in vivo* ^2^ conditions. Based on these results, we sought to evaluate whether bacteriophages could be considered a target for treating STEC infections. To this aim, we used GFP as an *in vitro* and *in vivo* reporter of phage dissemination based on a lysogenic *E. coli* C600ΔTOX:GFP strain and following bacteriophage induction. GFP expression was observed in liver, intestine and kidney of mice orally infected with the lysogenic strain and subsequently exposed to phage inducing conditions by oral administration with ciprofloxacin. Of particular relevance was the observation that chitosan, a natural polysaccharide polycationic polymer, exerted a direct inactivation effect on ϕΔTOX:GFP *in vitro* and drastically reduced the detection of fluorescence in mice orally infected with the lysogenic *E. coli* C600ΔTOX:GFP strain. Our findings indicate that chitosan possesses strong anti-bacteriophage effects *in vitro* and probably also *in vivo,* as demonstrated with the lysogenic *E. coli* C600ΔTOX:GFP strain. This positively charged polymeric polysaccharide has been reported to inhibit other bacteriophages and probably acts through electrostatic interactions with negatively charged capsid proteins ^6^. Based on these effects we propose that chitosan could be a viable alternative for the treatment of STEC infections. Chitosan is already used in food and medicine, and it is harmless to humans, making it a cheap and safe option for this application.

The same chitosan protective effects were also observed *in vivo* based on mice infected with the wild-type EHEC EDL933W strain which is lysogenic for the same bacteriophage used to generate the *E. coli* C600ΔTOX:GFP strain. The fact that only partial protection was observed in mice infected with the *E. coli* C600ΔTOX:GFP strain and subsequently treated with chitosan may be due to the short half-life of the compound ^12^.

Altogether, these findings suggest a paradigm change on the role of bacteriophages in STEC infections, indicating that these bacteriophages have a pivotal role on the development of HUS. The present observations further suggest that prophylaxis and treatment of human bacterial infections carrying virulence factors on lysogenic bacteriophages could require targeting of the bacteriophages instead of, or as well as, the bacteria and toxins involved.

## Data availability

F1000Research: Dataset 1. Induction of ϕΔTOX:GFP strain by ciprofloxacin and chitosan effect, 10.5256/f1000research.3718.d34269 ^13^
