# Supplementary material for: Role of bacteriophages in STEC infections: new implications for the design of prophylactic and treatment approaches
Source: F1000Res. 2014 Aug 26;3:74. Originally published 2014 Mar 18. [Version 2] doi: 10.12688/f1000research.3718.2 (PMC4288416; doi:10.12688/f1000research.3718.2)
Supplement: Peer review history

# Peer review: Referee response for version 2

**DOI:** 10.5256/f1000research.5299.r5927

**Published:** 2015-01-05

**Authors:** Mikael Skurnik

**Affiliations:** 1 Department of Bacteriology and Immunology, University of Helsinki, Helsinki, Finland

First of all I am sorry for the long delay in my getting back to this evaluation. While the authors have replied adequately to some of my comments there are some that are not. I will deal them below.

**Major points:**

**Point 1.** It is not enough to write the data of the dose response experiments in the reply. The results of the dose response experiments should be integrated into the main article.

**Point 2. **Inclusion of the mouse experiment (Fig. 4) in the article is not acceptable and should be deleted completely. In addition to having relatively small number of mice per group, the use of both male and female mice in the experiments is not appropriate as the responses can vary between the sexes. This and the small numbers of mice could be the reason for non-consistent killing results. In addition, it is not clear either from the authors' reply of from the text and the figure, how many mice were actually in the experiment. The figure 4 indicates that 50 % of both mouse groups died. How many mice was 50%, one or two? I have hard time to believe that two non-treated mice both died after 72 hr and two chitosan-treated both after 168 hr after infection and at the same time two mice in both groups survived. Therefore the mouse experiments in my opinion do not have any significance to one or other direction and should not be included in the present article.

**Point 3.** While the both controls the authors used are OK, they don't control the effect of ciprofloxacin in the experiment. Chitosan could also neutralize ciprofloxacin and thereby rescue the bacterial growth. The E. coli C600 strain is very widely used strain and should be easy to get. Preferentially it should be from the same source from where the lysogenic strains were obtained. I have the strain also in my strain collection and I can send it to the authors if they don't find it elsewhere.

**Point 4. **OK

**Point 5. **While the response seems OK, I don't find anywhere in the article the results and the additional figure. These should be integrated to the article.

**Point 6.  **Even though the C600 strain is not invasive, bacteria may be taken up by endocytosis and thereby enter the organs. The authors should exclude this possibility. While it would be difficult to detect the bacteria from intestinal tissue, other organs should be sterile.If the authors cannot see any bacterial growth from the sterile organs, that would be a sufficient control.

**Point 7. **See point 2 above.

All previous **Minor points **were all addressed adequately.

Please check carefully the text on page 4 right column top paragraph.

I have read this submission. I believe that I have an appropriate level of expertise to confirm that it is of an acceptable scientific standard, however I have significant reservations, as outlined above.

# Peer review: Referee response for version 2

**DOI:** 10.5256/f1000research.5299.r6885

**Published:** 2014-12-29

**Authors:** Cristina Ibarra

**Affiliations:** 1 Laboratory of Physiopathology, University of Buenos Aires, Buenos Aires, Argentina

This is an interesting paper where the authors have examined the hypothesis that  the stx-carrying prophage upon induction *in vivo* are required for the development of HUS in the murine model. Eukaryotic host cells would transduce these phages, leading to increase of Stx production in the target organs. This would explain why very small numbers of bacteria are sufficient to cause HUS. The authors have also examined the anti-bacteriophage effects of chitosan *in vivo* and *in vitro* and demonstrated that chitosan has strong inhibitory effects on STEC bacteriophage and suggest that it could be a prophylactic for the prevention and treatment of HUS patients.

In general, the presented study is conducted straight forward, is written concisely and the applied methodology is adequate.

I recommend indexation of this article without changes.

I have read this submission. I believe that I have an appropriate level of expertise to confirm that it is of an acceptable scientific standard.

# Peer review: Referee response for version 2

**DOI:** 10.5256/f1000research.5299.r6090

**Published:** 2014-09-09

**Authors:** Raúl Raya

**Affiliations:** 1 Genetics and Molecular Biology, Centro de Referencia Para Lactobacilos (CERELA), San Miguel de Tucumán, Argentina

I consider the authors have satisfactorily answered all my comments and that the manuscript is acceptable.

Should the phrase:  “T *he fact that only partial protection was observed in mice infected with the E. coli C600ΔTOX:GFP strain and subsequently treated with chitosan may be due to the short half-life of the compound*.” instead be: “ *The fact that only partial protection was observed in mice infected with the wild-type EHEC EDL933W strain and subsequently treated with chitosan may be due to the short half-life of the compound*”?

The authors should indicate the concentration of DNAase used.

I have read this submission. I believe that I have an appropriate level of expertise to confirm that it is of an acceptable scientific standard.

# Peer review: Referee response for version 1

**DOI:** 10.5256/f1000research.3984.r4510

**Published:** 2014-04-17

**Authors:** Raúl Raya

**Affiliations:** 1 Genetics and Molecular Biology, Centro de Referencia Para Lactobacilos (CERELA), San Miguel de Tucumán, Argentina

The article written by Amorim *et al.* describes the anti-phage activity of chitosan on two variants (wild type and a derivative where the *stx* gene was replaced by the *gfp* gene) of the temperate Shiga-toxin producing phage EDL933W. The anti-phage activity was evaluated both *in vivo* and *in vitro*. The authors suggest that chitosan could be a viable alternative for the treatment of STEC infections.

**Major:**

**Phage Induction/anti-phage effects of chitosan/Figure 1:**

It seems that chitosan not only sequesters free-phage particles, but also stimulates the growth of uninduced cells (see induced cells treated with chitosan 2 hours post-induction reached higher final OD values). So, does chitosan inhibit the induction process of the temperate phage? Or, does chitosan also adsorb/inactivate ciprofloxacin? Even though in the Materials and Methods a “Bacteriophage inactivation assay” is described, no data is presented. A dose-response curve should be presented, to determine the phage binding (inactivation) capacity of chitosan.

**In vivo experiments:**

If the authors suggest that Stx phages, rather than bacterial cells, may be responsible for the development of the STEC infections, why they did not use purified phage in *the vivo* experiments? Does chitosan adsorb/inactivate the Shiga-toxins? If so, may it explain the delayed response observed in Figure 4 (“EDL933W plus chitosan”)?

**Minors:**

**Abstract:**

- Provide a reference after “… plasmids encoding Stx under control of the native bacterial promoter.”
- Change “ *E. coli* K12 strain” to “ *E. coli* C600 strain”.

 

**Materials and Methods:**

- Delete “… was generously provided by Dr. Luis Carlos de Souza Ferreira, LDV-USP, Brazil.”, since Dr. Ferreira is one of the authors of the manuscript.
- Check the sentence “…was generously provided by Dr. Alison Weiss”; it is repeated twice in the Materials and Methods, and also in the Acknowledgments.
- Change “This is a non-pathogenic phage…” to “This is a non-pathogenic cell…”
- Change “…complete DMEM medium” to “DMEM medium”. Or, if the “complete DMEM medium” contains 10% fetal bovine serum, change “…complete DMEM medium” for “…complete medium”.
- Please, indicate how DNA was harvested.
- Change “Tris pH8 50mM” to “Tris pH8 50 mM”.
- Change “BALB/c mice were bred…” to ““BALB/c and DBA-2 mice were bred…”
- Change “…under a 12-h light-dark…” to “…under a 12-hour light-dark…”.
- Delete the sentence “Two-month old BALB/c mice … and GFP dissemination by IVIS was analyzed”. It is redundant.

 

**Results:**

- Figure 1B: Should “Bacteriophage/ml” be “PFU/ml”? Why phage titers are so low?
- Figure 1A: Change “hs” for “hours” or “h”.
- Figure 2: Lanes 5 and 6 should read: “positive PCR control” and “negative PCR control”, respectively. In lane 78, indicate in the figure the kb values of the ladder.
- Delete “viable” in “viable phages”. Were phages transduced or adsorbed to mammalian cells?
- The sentence “Mice were orally challenged with a wild-type EDL933W” is not correct, since there was a direct delivery of bacterial cells into the stomach of mice.

**Discussion:**

 

- Check “deliv-ery”

I have read this submission. I believe that I have an appropriate level of expertise to confirm that it is of an acceptable scientific standard, however I have significant reservations, as outlined above.

# Response

**Published:** 2014-08-01

**Authors:** Leticia Bentancor

**Affiliations:** UNQ, Argentina

**Phage Induction/anti-phage effects of chitosan/Figure 1:**

The higher final OD values determined on the cells treated with chitosan 2 hours post-induction, versus the OD value determined on un-induced cells, is not statistically significant. However, we tested if chitosan is capable of inactivating ciprofloxacin. Ciprofloxacin and chitosan were incubated for 10 minutes at room temperature with chitosan at 5mg/ml. After pre-incubation, the mix was used to induce bacteriophage excision. To see a more significant effect on bacteriophage excision, the induction was incubated overnight. The OD value measured showed a non-significant difference between non-induced culture and induced culture with the antibiotic pre-incubated with chitosan (the experiment was performed in triplicate).

On the other hand, purified bacteriophages were incubated with chitosan, and bacteriophage inactivation was observed with a lysis plaques assay. Bacteriophages were incubated for 10 minutes at room temperature with chitosan at 5mg/ml. After incubation, bacteriophage inactivation was evaluated. The bacteriophage solution containing a titer of 4x10 ^3^pfu/ml was 100% inactivated after chitosan incubation. The assay was performed in triplicate. This result showed the capacity of chitosan to inactivate bacteriophage *in vitro*.

Also, we did a dose-response curve of chitosan. We used 5mg/ml, 2.5mg/ml, and 1mg/ml of chitosan on purified bacteriophage solution. To evaluate it, the bacteriophage solution was incubated for 10 minutes at room temperature with the different doses of Chitosan and the bacteriophage inactivation was evaluated with a lysis plaques assay. Chitosan at 1mg/ml lost the inactivation activity on the bacteriophages. Chitosan at 5mg/ml and 2.5 mg/ml showed a 100% efficiency on bacteriophage inactivation, however, 1mg/ml of chitosan showed a loss of inactivation, showing between 5-10% of bacteriophage inactivation. This experiment was performed in triplicate.

***In vivo*** **experiments:**

Chitosan was analyzed *in vitro* and *in vivo* on fDTOX:GFP. Inactivation of fDTOX:GFP was observed *in vitro* with a lysis plaques assay. On the other hand, a decrease of GFP was observed *in vivo* by IVIS. These results shown that chitosan has the capacity to inactive bacteriophages in absence of Shiga-toxins. A direct action of chitosan on Shiga toxin was not evaluated in this work since we do not have purified Stx2 for such experiments. The authors are working on murine infection with fStx2, but the results obtained will be part of a new publication.

**Abstract:**

- *Provide a reference after “… plasmids encoding Stx under control of the native bacterial promoter.”*
  The reference was provided. 
- Change “ *E. coli* K12 strain” to “ *E. coli* C600 strain”.
  The change was made.

**Materials and Methods:**

- *Delete “… was generously provided by Dr. Luis Carlos de Souza Ferreira, LDV-USP, Brazil.”, since Dr. Ferreira is one of the authors of the manuscript.*
  Answer:  We deleted “… was generously provided by Dr. Luis Carlos de Souza Ferreira, LDV-USP, Brazil.”
- *Check the sentence “…was generously provided by Dr. Alison Weiss”; it is repeated twice in the Materials and Methods, and also in the Acknowledgments.*
   We deleted the sentence in the sub-section “ *Transduction of eukaryotic cells*” of material and methods section. 
- *Change “This is a non-pathogenic phage…” to “This is a non-pathogenic cell…”*
  We changed “This is a non-pathogenic phage…” to “This is a non-pathogenic cell…”. However, this non-pathogenic cell produces the excision of a non-pathogenic phage.
- *Change “…complete DMEM medium” to “DMEM medium”. Or, if the “complete DMEM medium” contains 10% fetal bovine serum, change “…complete DMEM medium” for “…complete medium”.*
  Complete DMEM medium contains 10% fetal bovine serum, so, we changed for the second option “…complete medium”.
- *Please, indicate how DNA was harvested.*
  Cells were harvested using Trypsin-EDTA solution. After that, DNA was harvested from pellets by incubation with lysis solution described in material and methods. We included “by Trypsin-EDTA incubation” to clarify the procedure.
- *Change “Tris pH8 50mM” to “Tris pH8 50 mM”.*
  The change was made.
- *Change “BALB/c mice were bred…” to ““BALB/c and DBA-2 mice were bred…”*
   We changed “BALB/c mice were bred…”  for ““BALB/c and DBA-2 mice were bred…”
- *Change “…under a 12-h light-dark…” to “…under a 12-hour light-dark…”.*
  The change was made.
- *Delete the sentence “Two-month old BALB/c mice … and GFP dissemination by IVIS was analyzed”. It is redundant.*
  We have two different mouse models. First, we have the model used to analyze GFP dissemination in which we used two months old mice. Second, we have the model used to analyze protection effect in which we used immature mice. For this reason we clarify the model every time. Let me know if you consider that we need to delete the sentence   “Two-month old BALB/c mice … and GFP dissemination by IVIS was analyzed”.

**Results:**

- *Figure 1B: Should “Bacteriophage/ml” be “PFU/ml”? Why phage titers are so low?*
  Bacteriophage/ml was changed to PFU/ml as reviewer suggested. See below. It is true that bacteriophage titers are low. An optimization for bacteriophage purification was done to obtain a higher titer of bacteriophage. The antibiotics used to induce C600DTOX:GFP was selected as an alternative for mitomycin C. The efficiency of bacteriophage induction is strain dependent.  Zhang and collaborators reported a titer equal to 1,3x10 ^5^ pfu/ml using ciprofloxacin but they used pathogenic strain *E. coli* O157:H7.  We also observed a higher titer inducing the EDL933W strain, for this reason we suppose that the low titer observed is dependent on the strain used.
- *Figure 1A: Change “hs” for “hours” or “h”.*
   The change was made.
- *Figure 2: Lanes 5 and 6 should read: “positive PCR control” and “negative PCR control”, respectively. In lane 78, indicate in the figure the kb values of the ladder.*
  The changes were made.
- *Delete “viable” in “viable phages”. Were phages transduced or adsorbed to mammalian cells?*
  We deleted “viable” in “viable phages”. In this context, phages purified from tissue were detected by lysis plaque assay.
- *The sentence “Mice were orally challenged with a wild-type EDL933W” is not correct, since there was a direct delivery of bacterial cells into the stomach of mice.*
   The sentence “Mice were orally challenged with a wild-type EDL933W” was change by “Mice were intragastrically infected with a wild-type EDL933W”.

**Discussion:**

- *Check “deliv-ery”*
  We did not find deliv-ery in the Discussion section.

# Peer review: Referee response for version 1

**DOI:** 10.5256/f1000research.3984.r4170

**Published:** 2014-03-31

**Authors:** Mikael Skurnik

**Affiliations:** 1 Department of Bacteriology and Immunology, University of Helsinki, Helsinki, Finland

The paper by Amorim *et al. *deals with the role of bacteriophages in STEC-infections. The authors have earlier demonstrated that the *stx *genes can be expressed within eukaryotic cells, provided the prophage-carried *stx-*DNA is introduced there in naked form, i.e., in transfected plasmids. In the present work, the authors wanted to test/prove the hypothesis that the *stx-*carrying prophage upon induction *in vivo *could contribute to the toxin production. They also tested whether the polysaccharide chitosan has anti- *stx* phage effect. I have some major and minor points:

**Major**

1. **Bacteriophage inactivation assay:** The experimental design of the bacteriophage inactivation assay uses only one concentration of chitosan. To demonstrate specificity, dose dependence should be demonstrated. In addition, the *in vivo* dose of chitosan was not indicated in the methods section (100 µl/mouse of 5 ml/ml chitosan was given orally to mice as indicated in the **Effect of chitosan *in vivo*** section.
2. The mouse experiments were performed with too small a number of mice.
3. Figure 1A of growth curves is missing a crucial control. What happens to E. coli C600 under the ciprofloxacin treatment?
4. Figure 1B: The lack of the 4 hr column in chitosan 4h post-induction does not seem logical to me. There should be a ca 6000 PFU/ml column similar to that one in the induced 4hr sample. This discrepancy should be explained.
5. Figure 2: the PCR experiment does not provide evidence of transduction. The definition of transduction is that DNA moves from one cell to another. PCR detects the phage DNA either free in the cell cytoplasm or packed in endocytosed phage particles. Therefore, the authors need to demonstrate that infective phage particles disappear from infected cells. The experiment also does not exclude the possibility that phage particles are just adsorbed on the cell surface.
6. The experiment reported in figure 3 should also include bacterial counts from the organs as it is very likely that live E. coli bacteria, after a massive dose of 10 ^13^ bacteria per mouse, end up in the organs. Therefore the authors should demonstrate that the GFP response is not from bacteria infected by the GFP-phages.
7. The Figure 4 experiment was performed with only 4 mice. Such an experiment should not be shown in a publication. In addition, different chitosan doses should be tested here also.

**Minor**

1. **Introduction, paragraph 3:** This statement on the low number of bacteria during infection should be backed up with a reference.
2. **Materials and Methods:** Dr Alison Weiss is thanked twice for same strain. One time should be enough. In addition, in the acknowledgements she is thanked a third time. The bacterial strain designation in the latter is given differently than elsewhere in the text.
3. **Transduction of Eukaryotic Cells:** C600ΔTOX:GFP is a bacterial strain, not a non-pathogenic phage.
4. **EHEC infection:** The final concentration of CFU/100µl/mouse needs revision.
5. **Figure 2 legend:** The path the sample takes in the gel is called the lane, not line.

I have read this submission. I believe that I have an appropriate level of expertise to confirm that it is of an acceptable scientific standard, however I have significant reservations, as outlined above.

# Response

**Published:** 2014-08-01

**Authors:** Leticia Bentancor

**Affiliations:** UNQ, Argentina

**Major:**

1. A dose-response curve of chitosan was done. We used 5mg/ml, 2.5mg/ml and 1mg/ml of chitosan on purified bacteriophage. To evaluate it, the bacteriophage solution was incubated for 10 minutes at room temperature with the different doses of chitosan and the bacteriophage inactivation was evaluated with a lysis plaques assay. Chitosan at 1mg/ml lost the inactivation activity on the bacteriophages. Chitosan at 5mg/ml and 2.5 mg/ml showed a 100% efficiency on bacteriophage inactivation, however, 1mg/ml of chitosan showed a loss of inactivation, showing between 5-10% bacteriophage inactivation. This experiment was performed in triplicate.
  The effect of chitosan *in vivo* was evaluated using a final concentration of 5mg/ml of chitosan solution. Each mouse received 100ml, so, the dose used was 500mg/mouse.
  The Material and Methods section was changed as follows:
  “Immature male and female DBA-2 infected, as described previously, were treated with 100ml of a chitosan solution at a concentration of 5 mg/ml (500 mg of chitosan per mouse) was orally administrated 2 hours after infection and survival was observed.”
2. The experiment was shown as a preliminary result and this work it is a short communication. The model used has some experimental problems for the ages of mice used. The experiment was started using 6 mice per group, but some mice died after inoculation and not for the infection. For this reason, we had shown only 4 mice per group. We repeat the experiment, and again we have the same problem, however, we can see the same partial effect of chitosan *in vivo*. To further analyze the effect observed, we will report the results on a new publication with more details. 
3. The controls we used were:
  1. Non-induced *E. coli* C600DTOX:GFP (as a negative control) in which we can observe the normal growth of bacteria without bacteriophage induction
  2. Induced *E. coli* C600DTOX:GFP (as a positive control) in which we can observe how bacteriophage induction affect the growth of bacteria.

If you are thinking about *E. coli* C600 in absence of lisogenic fDTOX:GFP, we do not have access to this strain. But, we think that the controls used are well done. As an observation, we can said that no significant change in the growth was observed on *E. coli* Y1090 used for bacteriophage titration assay.

1. The observation is right; we made an error in the graph. The values were checked and the correct value was added to the new graph.
2. We agree with the definition of transduction, it is the process by which DNA is transferred from one cell to another by a virus. In our previous paper, we used the same definition to evaluate the capacity of fDTOX:GFP to transduce macrophages. In this case, we observed GFP expression and we concluded that macrophages were transduced by fDTOX:GFP. In this report, we did a different approach and we use PCR to detect bacteriophage DNA inside the cell.
  After your opinion, we did two assays. First, bacteriophages inside the cell were analyzed for titration assay. Second, as a preliminary data, fStx2 was used to transduce Vero cells, as a representative Stx2-susceptible cell line. 
  Infective bacteriophage particles were not detected on transduced cells. The assay was made using lysis plaque assay of cellular extracts.
**Results:**
  In order to analyze the transduction by an additional method, Vero cells were transduced with fStx2 and cytotoxicity induced by Stx2 was evaluated by microscopy. Vero cells transduced with fStx2 ( panel A) showed a similar cytotoxicity to that of cells incubated with 1 CD50 of purified Stx2 ( panel B). No cytotoxic effects were observed in non-treated Vero cells ( panel D). Vero cells transduced with a M.O.I. = 0,0625 did not shown cytotoxic effect, demonstrating the specificity of the effect observed by fStx2 ( panel D).
**Materials and Methods:**
** **
*In vitro* *evaluation of the capacity of Bacteriophage 933W to transduce Vero cells*.
*E. coli* EDL933W (ATCC 43895) was used to purify fStx2. *E. coli* EDL933W strain was grown in Luria Broth (LB) overnight (ON) at 37°C under agitation. The ON culture was diluted to OD600nm = 0.1 in LB. Induction was carried out by adding ciprofloxacin to a final concentration of 40 ng/ml ^8 in main text^. Bacteria were incubated for 6 hours at 37°C under agitation. Cultures were then centrifuged at 5000 rpm for 15 minutes. The bacteriophage-containing supernatant was filtered with 0.2 mm filters, precipitated and purified. Briefly, supernatant was incubated on ice with a PEG-8000/NaCl solution for 30 minutes. After that, the solution containing bacteriophages was centrifugated and washed. The pellet was resuspended in STE buffer (1ml of Tris pH8, 0,2ml of 0,5M EDTA, 2ml of 5M NaCl, water up to 100ml). Phages at a multiplicity of infection (M.O.I) equal to 1 were added to Vero cells. Transduction of Vero cells was enhanced by centrifugation at 1000 x g for 10 min at room temperature. After 24 hours post transduction, cells were examined by microscopy using Nikon Eclipse TE2000 (NIS-Elements imaging software) equipped with a CCD camera. Dilutions of fStx2 were made to demonstrate specificity. Vero cells were incubated with purified Stx2 as positive control.
**Additional figure**. *In vitro* evaluation of the capacity of Bacteriophage 933W to transduce Vero cells. **A**. Vero cells transduced with fStx2 (M.O.I. = 1). **B**. Vero cells incubated with purified Stx2. **C**. Vero cells transduced with a M.O.I. = 0,0625. **D**. Vero cells with not treatment.
3. *E. coli *C600DTOX:GFP is not an invasive bacteria. Also, *E. coli* O157:H7 is a non invasive strain; for this reason we do not check for bacteria in organs. Bacteria were checked only on lungs samples, just to see if the inoculation was right. Bacteria were not detected in lungs. The dose used was selected after a previous experiment in which we evaluated the sensibility of IVIS in our system. GFP is not the best fluorescent protein for IVIS system; so, we needed to use a high dose of bacteria.  As we described in this work, bacteriophages were detected by lysis plaques assay in intestine homogenates and blood samples of infected mice. It is important to do a highlight in the case of intestine sample, as it is very difficult to find *E. coli* C600DTOX:GFP. First, because the huge amount of bacteria present in the sample, and also, because the bacteria lysis induced by bacteriophage excision.
4. The experiment was shown as a preliminary result and this work it is a short communication. The model used has some experimental problems for the ages of mice used. The experiment was started using 6 mice per group, but some mice died after inoculation and not for the infection. For this reason, we had shown only 4 mice per group. We repeat the experiment, and again we have the same problem, however, we can see the same partial effect of chitosan *in vivo*. To further analyze the effect observed, we will report the results on a new publication with more details. 

**Minor:**

1. The statement “…very small numbers of bacteria are sufficient to induce HUS in humans…” is taking the data published recently, in which the authors demonstrated that a concentration of Stx2 as low as 10 fM is able to induce ribosome damage and to modulate selected cell signaling pathways that change cellular functions. If 10 fM of Stx2 is enough, very small numbers of bacteria should be sufficient to induce HUS (Petruzziello-Pellegrini & Marsden, 2012).
2. We deleted the sentence “…was generously provided by Dr. Alison Weiss” in the sub-section “Transduction of eukaryotic cells” of material and methods section.
3. We changed “This is a non-pathogenic phage…” to “This is a non-pathogenic cell…”. However, this non-pathogenic cell produces the excision of a non-pathogenic phage.

1. The dose is correct. We used a dose of 3 × 10 ^12^ CFU/mice in a volume of 100 ml.
2. Line was changed for Lane.
